# Development of an optimum photon beam model for head‐and‐neck intensity‐modulated radiotherapy

**DOI:** 10.1120/jacmp.v8i4.2711

**Published:** 2007-11-08

**Authors:** Gareth J Webster, Carl G Rowbottom, Ranald I Mackay

**Affiliations:** ^1^ North Western Medical Physics Christie Hospital NHS Trust Manchester United Kingdom

**Keywords:** IMRT, TPS, commissioning, verification, rounded leaf ends

## Abstract

Intensity‐modulated radiotherapy (IMRT) for complex sites such as tumors of the head and neck requires a level of accuracy in dose calculation beyond that currently used for conformal treatment planning. Recent advances in treatment planning systems have aimed to improve the dose calculation accuracy by improving the modeling of machine characteristics such as interleaf leakage, tongue and groove, and rounded multileaf collimator (MLC) leaf ends. What is uncertain is the extent to which these model parameters improve the agreement between dose calculation and measurements for IMRT treatments.

We used Pinnacle version 7.4f (Philips Medical Systems, Andover, MA) to carry out optimization of additional photon‐beam model parameters for both an Elekta Precise (Elekta, Stockholm, Sweden) and a Varian (Varian Medical Systems, Palo Alto, CA) linear accelerator (LINAC). One additional parameter was added to the beam models in turn, and associated models were commissioned to investigate the dosimetric impact of each model parameter on 5 clinical head‐and‐neck IMRT plans. The magnitude and location of differences between the models was determined from gamma analysis of the calculated planar dose maps. A final model that incorporated all of the changes was then commissioned. For the Elekta Precise, the impact of all the changes was determined using a gamma analysis as compared with measured films.

Cumulative differences of up to more than 3%/3 mm were observed when dose distributions with and without all of the model changes were compared. Individually, for both LINACs, the addition of modeling for the rounded MLC leaf ends caused the most dramatic change to the calculation of the dose distribution, generating a difference of 3%/3 mm in up to 5% of pixels for the 5 patient plans sampled. The effect of tongue‐and‐groove modeling was more significant for the Varian LINAC (at 1%/1 mm, mean of 25% of pixels as compared with 5% of pixels with the Elekta Precise LINAC). The combined changes to the Elekta model were found to improve agreement with measurement.

Current commercially available treatment planning systems offer accuracy sufficient for clinical implementation of head‐and‐neck IMRT. For this treatment site, the ability to accurately model the rounded MLC leaf ends has the greatest affect on the similarity of the calculated dose distribution to measurements. In addition, for the Varian LINAC, modeling of the tongue‐and‐groove effect was also advantageous.

PACS numbers: 87.53.‐j, 87.53.Bn, 87.53.Tf

## I. INTRODUCTION

The delivery of intensity‐modulated radiotherapy (IMRT) may require greater accuracy in the beam model used for calculation of the dose distribution than has traditionally been demanded for conformal radiotherapy techniques. The increased complexity of IMRT plans can result in a significant proportion of the dose in high‐dose regions being a summation of dose contributions from penumbra and out‐of‐field regions from several individual beam segments. Dose inaccuracies that would be accepted in low‐dose regions can become very important when they contribute to high‐dose regions—for example, close to critical structures. The case of penumbrae is particularly relevant, because a slight error in penumbral position could cause a marked change in the dose at a particular point.

A study by Schwarz et al.^(1)^ investigated the reliability of a standard treatment planning system (TPS) in calculating an IMRT dose distribution. Those investigators first verified that two commercial TPSs fulfilled the dose calculation accuracy criteria for conventional treatment techniques, as reported by the American Association of Physicists in Medicine and others.^(2)^ They then used the two beam models to calculate the dose distribution to a series of prostate and head‐and‐neck IMRT plans for comparison with measured segment doses, demonstrating good dose agreement (<3%) for points in the center of 95% of segments, but errors of up to 40% (more than 5% of isocentric dose) when measured beneath the jaws. The discrepancies between the two systems were compounded by the calculated values being either side of the measured values. Their work confirms that the accuracy criteria required in beam models for conventional radiotherapy is insufficient for the clinical implementation of IMRT, because a critical dose to, for example, the spinal cord could be significantly underestimated if it is partly the summation of out‐of‐field or penumbral doses from several segments.

Modern commercial TPSs form mathematical representations of the radiation beam based on a number of variable parameters that reflect particular aspects of the linear accelerator (LINAC). The Philips Pinnacle TPS (Philips Medical Systems, Andover, MA) has recently been upgraded, incorporating a number of new features into the modeling software^(3)^ to improve on the limitations of the previous release.^(4)^ These include an improved auto‐modeling facility that may remove the previous subjectivity from the modeling process, and refinements to the model itself, such as tongue‐and‐groove width, interleaf leakage, independence of *x* and *y* jaw transmissions, modeling of the flattening filter as a radially‐symmetric arbitrary profile rather than as a cone, and modeling of rounded MLC leaf ends and leaf offsets.

Test patterns have been developed to allow accurate determination of several of these modeling parameters by iterative adjustment to agreement with measured profiles.^(3)^ Values for the groove width and MLC transmission can be determined by matching profiles from measurement and from the TPS. Similar tests were carried out to determine the curvature of rounded MLC leaf ends and interleaf leakage. The resulting beam model was applied to a clinical head‐and‐neck IMRT dose distribution, using film measurement to demonstrate good agreement (mostly within 4%/4 mm), with the error partly attributable to over‐response of the film in low‐dose regions. No attempt was made to quantify the clinical or dosimetric benefits of accurately determining these parameters and so did not confirm their clinical impact on the dose distribution.

Williams and Metcalfe^(5)^ describe, for Pinnacle v7.4f, the effect on penumbra width and profile modeling of changes in specific modeling parameters such as leaf‐end radius of curvature and intraleaf transmission. They applied an optimized model to a clinical IMRT field to demonstrate the effects of tongue‐and‐groove width and rounded leaf‐end modeling on a sample IMRT plan. They used gamma analyses (2 mm/3%) to quantify these effects for the two software versions as compared with a dose‐to‐water image obtained with an electronic portal imaging device and found improved accuracy to 2.2% from 7.0% pixel failure with the new version, although how much of the improvement was attributable to the improved modeling of the rounded MLC leaf ends and how much to the addition of the groove width to the model was unclear. Absolute dose calculation also demonstrated improved accuracy as compared with measurement.

Although much work has been done to optimize current beam modeling facilities and to move toward accurate measurement of the required parameters, the necessary work—in terms of its effect on clinical plans—has yet to be quantified. The aim of the present study was to evaluate the dosimetric impact of the improvements to the photon beam model description within Pinnacle and to identify the parameters to which the plans are most sensitive (warranting the most attention and being less amenable to compromise when modeling). We aimed to assess the individual impact of each of the previously described parameters on a series of clinical plans so as to determine which ones have a significant clinical impact. The study was carried out for both an Elekta Precise and a Varian 600C/D LINAC. Head‐and‐neck IMRT plans were chosen, because they represent more complex cases, in which the impact of the experimental changes is likely to be greater. The intention was that this work provide valuable information on which model parameters are required to be accurately modeled for IMRT treatments and the required sophistication of the beam modeling software.

## II. MATERIALS AND METHODS

A thorough explanation of the beam modeling process and the sequence followed by the Pinnacle TPS auto‐modeling software has been given by Starkschall et al.^(6)^ Although the modeling software has evolved since that publication, the methodology and most of the parameters used have remained unchanged. Only the modifications are described here.

**Figure 1:**
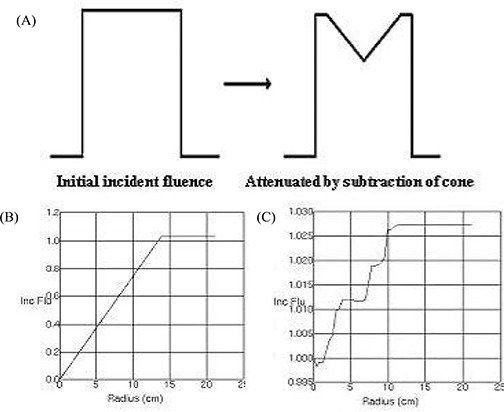
(A) A radially symmetric volume is subtracted from the initial fluence to account for the flattening filter. It can be (B) a uniform cone, or (C) the Pinnacle v7.4f treatment planning system provides the option of an arbitrary profile.

The flattening filter attenuates and hardens the beam to a greater extent in the beam's center. The attenuation has therefore previously been modeled as a subtraction of an inverted cone from the beam fluence, where the depth and radius of the cone can be specified. The attenuation can now be modeled as an arbitrary profile with radial symmetry. Fig. 1 illustrates both approaches.

The presence of an MLC can be designated by the user, whereupon a range of associated parameters, such as the tongue‐and‐groove width and interleaf leakage, are now available for definition. In regions below a full MLC leaf, a user‐defined MLC transmission factor is used to appropriately attenuate the initial beam intensity to that point. For regions at the field edges affected by the tongue‐and‐groove effect, the intensity is reduced by half of that to the full leaf, and for regions between two adjacent leaves, the user‐defined leakage value applies a correction factor to the initial intensity. For regions beneath the jaws, the intensity is attenuated according to the appropriate jaw transmission factor, which can now be assigned separately for the *x* and *y* jaws, an improvement on previous software releases, when an average value was required.

For regions underneath the leaf tip, the radius of curvature of the rounded MLC leaf tip, leaf offset position, and the effective attenuation coefficient are used to calculate the relative transmission through the leaf tip compared with that through the full leaf, as described in detail by Cadman et al.^(3)^ In the present work, the radius of curvature of the rounded MLC leaf ends and the leaf offset table were initially taken from values recommended by the Pinnacle planning system. The leaf offset table was then adjusted to better fit the measured data.

The individual impact of the various additions to the beam modeling facility were evaluated by commissioning 7 beam models, each with additions to a baseline model derived by the auto‐modeling software:

- Model i: an original baseline model, including none of the enhancements to the beam model
- Model ii: incorporates only the tongue‐and‐groove effect, using the recommended leaf width of 0.1 cm

- Model iii: incorporates 2% interleaf leakage
- Model iv: allows for the independence of *x* and *y* jaw transmissions, with optimized values (*x* transmission of 0.08, *y* transmission of 0.02) as compared with a value used in the baseline model (*x* and *y* transmission of 0.02)
- Model v: improves modeling of the flattening filter, using a radially‐symmetric arbitrary profile
- Model vi: accurately models the rounded MLC leaf ends and optimized leaf offsets
- Model vii: a combined model that incorporates all the modifications

**Figure 2:**
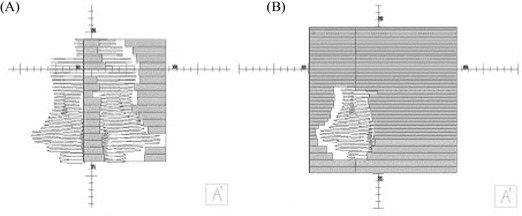
Typical segments defined for delivery on (A) the Elekta Precise (Elekta, Stockholm, Sweden) linear accelerator, in which the jaws track the leaf positions, and (B) the Varian 600C/D (Varian Medical Systems, Palo Alto, CA) linear accelerator, in which the jaws remain fixed as the leaf‐defined aperture moves between segments.

The beam model investigated on the two LINACs involved a 6‐MV beam. The present work should highlight any differences in the effects of the modeling changes between the two LINACs—changes that would likely be attributable to the changes in the IMRT segment delivery process illustrated in Fig. 2. On the Elekta machine, the jaws track the leaf motion for each segment, but on the Varian, the jaws remain fixed throughout beam delivery. As a result, for Varian delivery, almost all segments are defined by the rounded leaf ends and the leaf edges. The radius of curvature of the rounded leaf ends is set to 15 cm for the Elekta and to 12 cm for the Varian, in line with manufacturer‐recommended values. Because the jaws always remain outside the target volume for the Varian, model iv was omitted for that machine.

Each model (i – vii) was commissioned, and the resulting dose distribution calculated on a 2‐mm dose grid for a series of 5 head‐and‐neck IMRT patient plans on a semi‐anthropomorphic Perspex head‐and‐neck phantom that is routinely used in the verification of clinical head‐and‐neck IMRT treatments. To be able to examine the effect of these changes in regions of high dose, low dose, high dose gradient, and at a distance from the central axis, coronal dose planes were acquired through the phantom at the level of the spinal cord on Pinnacle. In‐house gamma analysis software written in IDL^(7)^ was then used to calculate the location and extent of any changes to the dose distribution resulting from the individual (ii – vi) and combined (vii) model changes. Each plan used 5 IMRT fields at 6 MV with a total of 65 – 82 segments and was planned according to the departmental protocol for the Parsport trial, which delivers a dose of 65 Gy to the tumor clinical target volume and involved nodes and 54 Gy to the elective nodes, and which attempts to spare the contralateral parotid.

To confirm that the changes to the beam model resulted in improved dose calculation accuracy, the baseline (i) and modified (vii) models for the Elekta were then used to calculate individual beam fluence maps for comparison with measured Kodak extended dose range (EDR2) film for the same cohort of patients for every field. This analysis was conducted using routine clinical practice, wherein each fluence map is analyzed with the 100% isodose normalized to fall within the high‐dose region. Similar work has been done for a single IMRT field,^(5)^ but has not been established over a number of clinical fields. Because access to film processing equipment was discontinued during the study, a comparison of the Varian model with film was not carried out.

## III. RESULTS

**Figure 3:**
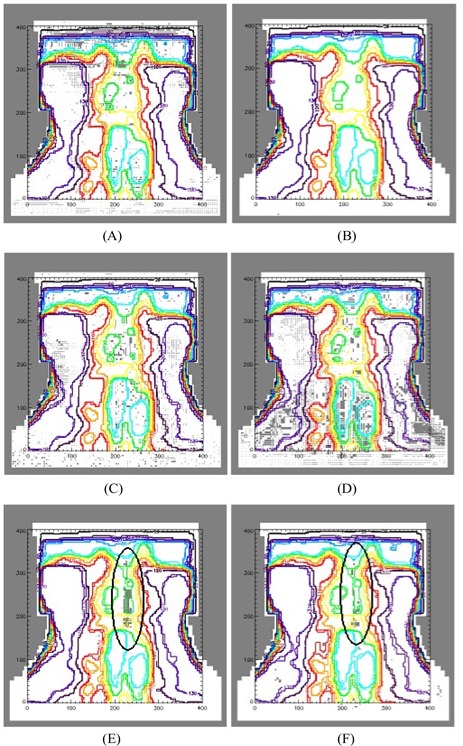
Typical gamma evaluation maps for coronal slices at the level of the spinal cord as compared with the baseline model: (A) tongue‐and‐groove width at 1%/1 mm; (B) interleaf leakage at 1%/1 mm; (C) independent jaws at 1%/1 mm; (D) arbitrary profile for flattening filter at 1%/1 mm; (E) full multileaf collimator model at 3%/3 mm, with circled region of pixel failure; and (F) all changes at 3%/3 mm. Regions of pixel failure are shown in grey; white represents pixels within the defined tolerance. The 80% isodose is shown in sky blue and was selected to include doses above the approximate level of the spinal cord tolerance. The 20% isodose, which encompasses almost all of the dose distribution, is shown in black.

Fig. 3 illustrates the effect on the coronal dose distribution for the individual and collective modifications to the Elekta Precise beam model for a typical clinical head‐and‐neck IMRT plan copied to a verification phantom. Table 1 (Elekta Precise) and Table 2 (Varian 600C/D) present the gamma analysis results for the sample of 5 patients. The results in these tables are given as percentage pixel failures either within high‐dose regions (>80% isodose) or for the overall dose distribution (>20% isodose). The tolerances for the various parameters were chosen to highlight the extent and, in Fig. 3, the location of significant variation. (A common high or low tolerance would have caused some gamma maps to be either empty or saturated as a result.)

**Table 1:** Percentage of pixels failing to meet gamma analysis limits, as compared with the baseline model (i) for the Elekta Precise (Elekta, Stockholm, Sweden) linear accelerator^a^

	Isodose			
	80%		20%	
Model	Mean	SD	Mean	SD
At 1%/1 mm				
(ii) Tongue‐and‐groove effect	4.9	1.2	5.9	1.8
(iii) Interleaf leakage	0.0	0.0	0.1	0.1
(iv) *x* and *y* jaw transmission	3.8	2.1	4.0	1.9
(v) Arbitrary flattening filter profile	12.6	3.7	11.8	3.7
At 3%/3 mm				
(vi) Full MLC model	1.5	1.0	1.6	0.9
(vii) All changes	1.6	1.0	1.4	0.8

a For models ii – iv, changes were observed only at the 1%/1 mm level, suggesting that the clinical impact of accurately modeling these parameters is limited, even for complex treatments such as intensity‐modulated radiotherapy for the head and neck. The larger changes to the dose distribution (apparent in model vi at 3%/3 mm) are attributable to the precise modeling of the shape and position of the rounded multileaf collimator (MLC) leaf ends. SD=standard deviation; MLC=multileaf collimator.

**Table 2:** Percentage of pixels failing to meet gamma analysis limits, as compared with the baseline model (i) for the Varian 600C/D (Varian Medical Systems, Palo Alto, CA) linear accelerator^a^

	Isodose			
	80%		20%	
Model	Mean	SD	Mean	SD
At 1%/1 mm				
(ii) Tongue‐and‐groove effect	25.5	2.0	22.8	2.7
(iii) Interleaf leakage	0.0	0.0	0.0	0.0
(iv) *x* and *y* jaw transmission	N/A	N/A	N/A	N/A
(v) Arbitrary flattening filter profile	13.8	3.5	12.0	1.9
At 3%/3 mm				
(vi) Full MLC model	0.3	0.1	0.3	0.2
(vii) All changes	0.8	0.4	1.1	0.8

a The independence of the jaws (iv) was not investigated for the Varian linear accelerator because the jaws always remain outside of the overall fluence. The tongue‐and‐groove effect (ii) was found to be more significant for the Varian than for the Elekta Precise (Elekta, Stockholm, Sweden). The effect of modeling the rounded leaf ends was diminished slightly, but remained significant.

SD=standard deviation; MLC=multileaf collimator.

The results suggest that the effect of interleaf leakage on planning would be negligible, except directly beneath the leaf gap. The independence of jaw transmission values in the Elekta model has a small impact at the 1%/1 mm level. Fig. 3(A) illustrates the addition to the Elekta model of the tongue‐and‐groove effect, which is found to have only a small effect on the dose distribution, predominantly in the superior region of the plan (mean of 4.9% of pixels altered at 1%/1 mm tolerance). For the Varian model, this effect is more widespread (24.6% of pixels altered at 1%/1 mm tolerance). The modeling of the flattening filter as a radially symmetric profile rather than a simple cone is found to have a consistent low‐level effect (<2%/2 mm) throughout the dose distribution for both LINACs. Fig. 3(E) has been calculated for the Elekta with a 3%/3 mm tolerance and demonstrates the significant impact of the accurate modeling of the rounded MLC leaf ends in high dose gradient regions such as the spinal cord (mean of 1.5% of pixels altered at 3%/3 mm tolerance). The close agreement of this image with the gamma image resulting from the addition of all modifications [Fig. 3(F)] confirms that accurate modeling of the rounded leaf ends is the dominant effect. This finding is also the case for the Varian model, although to a slightly lesser extent.

**Figure 4:**
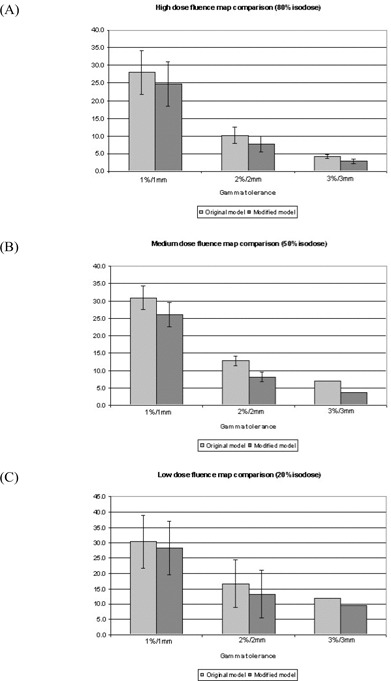
Comparison of the baseline (i) and fully modified (vii) models relative to measured fluence maps for 5 clinical head‐and‐neck intensity‐modulated radiotherapy plans (25 fields) for the Elekta Precise (Elekta, Stockholm, Sweden) linear accelerator, indicating the improved accuracy of the modified model.

Fig. 4 demonstrates that, for all dose levels, the combined effect of changes to the Elekta modeling parameters is to improve agreement with measured dose distributions. The improvements are significant (P<0.05) at the medium dose level for errors greater than 2%/2 mm, which is expected, because this level incorporates a higher proportion of high‐gradient areas in which the effects of errors in the penumbra are more prominent. Significant improvement of the larger errors (3%/3 mm) is also seen in the high‐dose region. Given the limited sample size, the improvements were not found to be as significant within the high‐ and low‐dose regions, probably because these regions are predominantly low‐gradient areas of the dose distribution.

## IV. DISCUSSION

The improvements to the beam modeling facilities in Pinnacle v7.4f have a statistically significant impact on the verification accuracy of IMRT plans for the head and neck. The addition of interleaf leakage values was found not to affect the resulting dose distribution, and values can be easily taken from a manufacturer's machine information. Accurate modeling of the tongue‐and‐groove effect was found to be more significant for the Varian 600C/D than for the Elekta Precise. That finding was expected, because for the Elekta delivery, the superior region contains a higher proportion of field edges defined by the combination of jaw and leaf edges as opposed to leaf edges alone. Other parts of the dose distribution result from the number of small, low‐weighted segments. Because of the static jaws in the Varian LINAC's delivery, almost all segments are defined by the leaf edge only, and so the introduction of the groove width has greater impact. The accurate modeling of the rounded MLC leaf ends is found to be of great importance to the dosimetric accuracy of the IMRT plan calculation. It is recommended that accurate modeling of rounded leaves be the focus of adjustments to the beam model when auto‐modeling has been carried out to set other parameters. The accuracy of the resulting beam model significantly and consistently improves agreement with measured fluence films.

Although this study focused on the Pinnacle TPS, its results highlight the need for any TPS to correctly model the penumbra of the MLC and jaws to enable good dosimetric agreement for head‐and‐neck IMRT treatments. The dose to critical structures such as the spinal cord is often defined by the superposition of multiple penumbrae in such treatments. The addition of the other parameters described is of limited clinical importance.

The ability of Pinnacle to accurately model the dose would be less accurate at higher energies (for example, 18 MV) because of relatively small field sizes and the problem of modeling electron transport at air–tissue interfaces in the head‐and‐neck region. Such modeling could be more accurate with Monte Carlo simulations, for example—although it is questionable whether treatment of head‐and‐neck sites would be improved by the use of such high photon energies.

## V. CONCLUSION

The accurate modeling of field penumbrae, particularly penumbrae defined by the rounded MLC ends, is of great importance to the commissioning of a TPS for the clinical implementation of head‐and‐neck IMRT and other sites for which the dose to critical structures is derived from the addition of many beam penumbrae. Current commercially available TPSs can offer sufficient accuracy for this treatment technique (within 3% – 4%),^(8)^ but the user must be aware of the system's limitations, both in terms of the dosimetric capabilities and the effects of the compromises made during the modeling process.

## References

[1] Schwarz M , Bos LJ , Mijnheer BJ , Lebesque JV , Damen EM . Importance of accurate dose calculations outside segment edges in intensity modulated radiotherapy treatment planning. Radiother Oncol. 2003;69(3):305–314. 14644490 10.1016/j.radonc.2003.09.002

[2] Fraass B , Doppke K , Hunt M , et al. American Association of Physicists in Medicine Radiation Therapy Committee Task Group 53: quality assurance for clinical radiotherapy treatment planning. Med Phys. 1998;25(10):1773–1829. 9800687 10.1118/1.598373

[3] Cadman P , McNutt T , Bzdusek K . Validation of physics improvements for IMRT with a commercial treatment planning system. J Appl Clin Med Phys. 2005;6(2):74–86. 15940214 10.1120/jacmp.v6i2.2083 PMC5723472

[4] Cadman P , Bassalow R , Sidhu NP , Ibbott G , Nelson A . Dosimetric considerations for validation of a sequential IMRT process with a commercial treatment planning system. Phys Med Biol. 2002;47(16):3001–3010. 12222862 10.1088/0031-9155/47/16/314

[5] Williams MJ , Metcalfe P . Verification of a rounded leaf‐end MLC model used in a radiotherapy treatment planning system. Phys Med Biol. 2006;51(4):N65–N78. 16467576 10.1088/0031-9155/51/4/N03

[6] Starkschall G , Steadham RE Jr , Popple RA , Ahmad S , Rosen II . Beam‐commissioning methodology for a three‐dimensional convolution/superposition photon dose algorithm. J Appl Clin Med Phys. 2000;1(1):8–27. 11674815 10.1120/jacmp.v1i1.2651 PMC5726162

[7] Low DA , Harms WB , Mutic S , Purdy JA . A technique for the quantitative evaluation of dose distributions. Med Phys. 1998;25(5):656–661. 9608475 10.1118/1.598248

[8] Ezzell GA , Galvin JM , Low D , et al. Guidance document on delivery, treatment planning and clinical implementation of IMRT: report of the IMRT subcommittee of the AAPM Radiation Therapy Committee. Med Phys. 2003;30(8):2089–2115. 12945975 10.1118/1.1591194

